# Protective effect of 1950 MHz electromagnetic field in human neuroblastoma cells challenged with menadione

**DOI:** 10.1038/s41598-018-31636-7

**Published:** 2018-09-05

**Authors:** Stefano Falone, Anna Sannino, Stefania Romeo, Olga Zeni, Silvano Jr. Santini, Roberta Rispoli, Fernanda Amicarelli, Maria Rosaria Scarfì

**Affiliations:** 1 Dept. of Life, Health and Environmental Sciences, University of L’Aquila, L’Aquila, Italy; 2 Institute for Electromagnetic Sensing of the Environment (IREA) - National Research Council (CNR), Naples, Italy; 3 Institute of Translational Pharmacology (IFT) - National Research Council (CNR), L’Aquila, Italy

## Abstract

This study aims to assess whether a 1950 MHz radiofrequency (RF) electromagnetic field could protect human neuroblastoma SH-SY5Y cells against a subsequent treatment with menadione, a chemical agent inducing DNA damage via reactive oxygen species formation. Cells were pre-exposed for 20 h to specific absorption rate of either 0.3 or 1.25 W/kg, and 3 h after the end of the exposure, they were treated with 10 µM menadione (MD) for 1 h. No differences were observed between sham- and RF-exposed samples. A statistically significant reduction in menadione-induced DNA damage was detected in cells pre-exposed to either 0.3 or 1.25 W/kg (P < 0.05). Moreover, our analyses of gene expression revealed that the pre-exposure to RF almost inhibited the dramatic loss of glutathione peroxidase-based antioxidant scavenging efficiency that was induced by MD, and in parallel strongly enhanced the gene expression of catalase-based antioxidant protection. In addition, RF abolished the MD-dependent down-regulation of oxoguanine DNA glycosylase, which is a critical DNA repairing enzyme. Overall, our findings suggested that RF pre-exposure reduced menadione-dependent DNA oxidative damage, most probably by enhancing antioxidant scavenging efficiency and restoring DNA repair capability. Our results provided some insights into the molecular mechanisms underlying the RF-induced adaptive response in human neuroblastoma cells challenged with menadione.

## Introduction

Recently, evidence has been accumulated that exposure to radiofrequency electromagnetic fields (RF-EMF) is able to induce beneficial effects *in vitro* and *in vivo*, by protecting bio-systems from damage derived from subsequent treatments with well known chemical or physical agents. These observations suggested that pre-exposure to non-ionizing radiation induces a phenomenon which is similar to the adaptive response (AR)^1^.

AR refers to the ability of cells or organisms to resist the damaging effects of a toxic agent when first pre-exposed to a lower dose of the same or of another agent. The low dose is usually referred to as adaptive dose (AD) and the high dose as challenge dose (CD). AR is a widespread phenomenon that has been observed in prokaryotes, yeast, plants and mammals^2,3^.

Most of the AR-related studies refer to ionizing radiation, but many different types of damaging agents, including alkylating agents, heat stress, oxidants and heavy metals have been reported to induce AR^4^.

Several action mechanisms have been proposed to explain the AR. Among them, the activation of numerous signalling pathways related to cell defences, the increase of antioxidant protection for a more efficient detoxification of free radicals and the up-regulation of DNA repair enzymes are thought to play a crucial role^3–5^.

Reactive oxygen species (ROS) are continuously produced by aerobic cells, and this represents a potential harm mainly due to the instability of such chemical species^6^. For this reason, cells are endowed with antioxidant mechanisms which rely on both non-enzymatic and enzymatic defence lines. The latter involves superoxide dismutases with different cellular localization (SOD1, mainly cytosolic; SOD2, mitochondrial), catalase (CAT) and glutathione peroxidase (GPX). Unfortunately, despite the protective barrier provided by antioxidant mechanisms, unscavenged ROS pose a threat to key macromolecules^7^. Hence, ROS-derived oxidative lesions may be repaired through the action of several proteins, among which the oxoguanine DNA glycosylase (OGG1) is emerging as a critical DNA repairing enzyme^8^.

In earlier studies, our research group evidenced that pre-exposure of mammalian cell cultures to RF-EMF at different frequencies, signals, and specific absorption rate (SAR) values was able to reduce DNA damage induced by a subsequent treatment with mitomycin-C (MMC)^9–12^ or X-rays^13^. Other research groups reported similar findings in animals and mammalian cells. Most of these studies were reviewed by Vijayalaxmi and co-workers^1^.

To provide more evidence on the phenomenon of RF-induced AR and its possible molecular mechanisms, in this study SH-SY5Y human neuroblastoma cells were pre-exposed for 20 hours to 1950 MHz, UMTS signal, and subsequently treated with menadione (MD). MD (2-methyl-1,4-naphtoquinone; also called vitamin K3) is a semi-quinone that undergoes one-electron reduction in the mitochondrial respiratory chain, followed by one-electron transfer to molecular oxygen, producing O_2_^·−^^14^. MD was chosen since it is a molecule largely used in the studies dealing with oxidative stress. Its metabolism involves redox cycling, resulting in the release of ROS^15^.

Following treatments, cell viability was assessed, along with DNA damage and transcriptional expression of enzymes related to antioxidant protection and DNA repair.

## Results

### Pre-Exposure to RF reduces the MD-induced DNA damage

**Figure 1:**
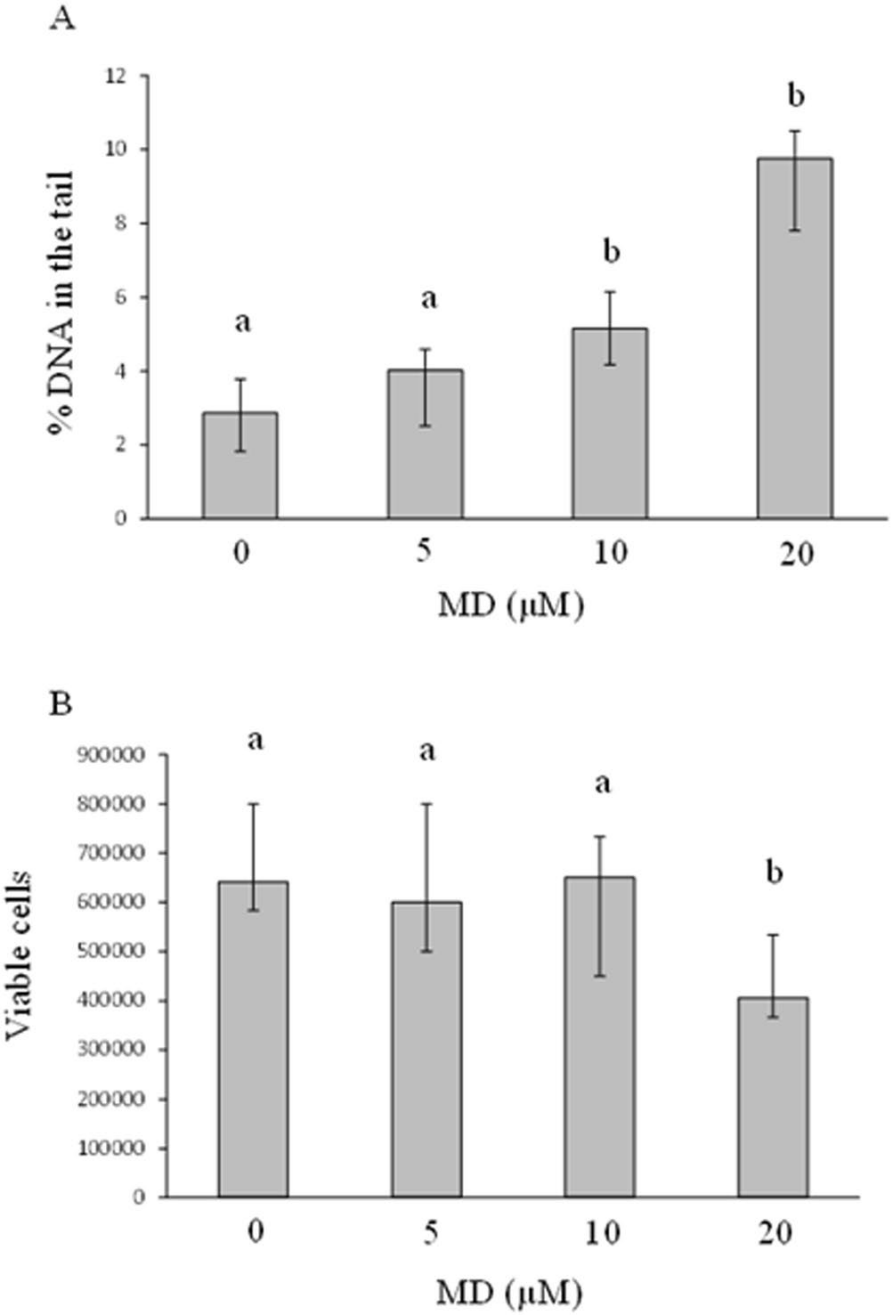
Medians with interquartile ranges of % DNA in the tail in 500 nuclei (panel A) and of viable SH-SY5Y cells (panel B) treated with different concentrations of MD for 1 h (at least six independent experiments). Same letters indicate no statistically significant differences (please, see main text for statistical details).

The results of at least six independent experiments aimed to identify the MD CD, are reported in Fig. 1, panel A. The non-parametric statistical analysis of data (Mann-Whitney U test) revealed that 5 µM MD was not enough to induce DNA damage, whereas 10 µM MD was sufficient to trigger statistically significant DNA oxidation (P < 0.001 *vs.* 0 µM MD). Similarly, the highest MD concentration (i.e., 20 µM) induced a statistically significant increase in % DNA in the tail, with respect to neuroblastoma untreated cells (P < 0.01), as well as a near-significant difference with respect to 10 µM MD (P = 0.063).

As shown in Fig. 1, panel B, which derived from at least six independent experiments, the statistical analysis revealed that both 5 µM and 10 µM MD did not change significantly the viable cell number, with respect to samples not treated with MD (i.e., 0 µM MD). Conversely, 20 µM MD reduced significantly the number of viable cells, as compared to MD-untreated cells (P < 0.05).

Taken together the results on viability and DNA oxidative damage, 10 µM MD concentration was used in all subsequent experiments.

**Figure 2:**
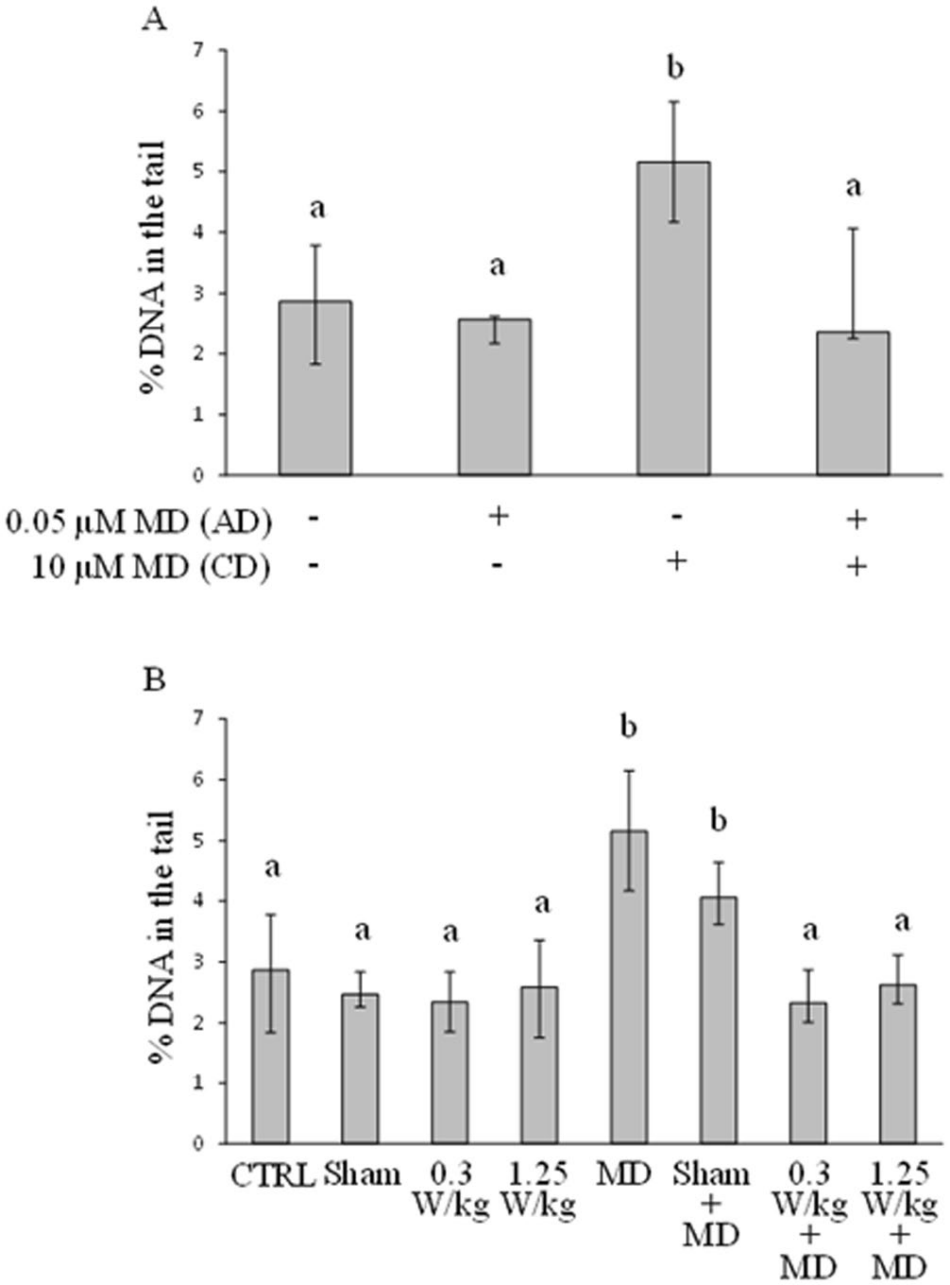
Medians with interquartile ranges of % DNA in the tail in 500 nuclei of SH-SY5Y cells: treated with 0.05 µM MD as adaptive dose (AD, given at 48 h after seeding) and 10 µM MD as challenge dose (CD, given at 71 h after seeding) (at least five independent experiments, panel A); untreated (CTRL); sham-exposed for 20 h (Sham); RF-exposed for 20 h at 0.3 W/Kg (0.3 W/Kg) or 1.25 W/Kg (1.25 W/Kg); treated with 10 µM MD for 1 h (MD); sham-exposed and treated with 10 µM MD for 1 h (Sham + MD); RF-exposed for 20 h and treated with 10 µM MD for 1 h (0.3 W/kg + MD; 1.25 W/kg + MD) (at least four independent experiments, panel B). Same letters indicate no statistically significant differences (please, see main text for statistical details).

The results of at least five independent experiments aimed to assess the responsiveness of SH-SY5Y toward an adaptation protocol where MD was used as adaptive dose (AD) and challenge dose (CD) are presented in Fig. 2, panel A. The statistics showed that the CD of menadione (i.e., 10 µM) was able to induce DNA damage with respect to MD-untreated cells (P < 0.001), whereas the pre-treatment of cells with the AD of menadione (i.e., 0.05 µM) was able to prevent such DNA-damaging effect (P < 0.05 AD+/CD+ *vs.* AD−/CD+). In all cases, viability was not affected by treatments (data not shown).

The results of four independent experiments aimed to assess the capability of RF given as AD to induce AR-resembling phenomenon, are reported in Fig. 2, panel B. The analyses showed that the sham-exposure did not affect significantly DNA damage level. Moreover, RF exposure at both SARs did not change significantly the level of oxidatively damaged DNA, with respect to sham-exposure. The rank sum test also revealed that MD increased significantly DNA damage, both in presence (P < 0.001) and absence (P < 0.05) of sham exposure. Interestingly, RF at both SAR levels prevented the MD-induced increase of oxidatively damaged DNA, with respect to both MD and Sham + MD (P < 0.05), with no statistically significant difference between the two SARs. Cell viability was unaffected for all the treatments considered (data not shown).

### Pre-Exposure to RF prevents MD-induced transcriptional changes of antioxidant and DNA repair enzymes

The results of comet assay showed no differences between control cultures kept in standard incubator and sham-exposed ones. Since sham-exposed cells experienced the very same environmental conditions as the exposed ones (except for RF exposure), the former were considered as the most appropriate reference control in all experiments on gene expression analysis. In addition, as no statistically significant difference in % DNA in the tail was found between cultures exposed to 0.3 W/kg and those exposed to 1.25 W/kg, all RT-PCR analysis were performed on samples exposed to the lowest SAR value (0.3 W/kg).

**Figure 3:**
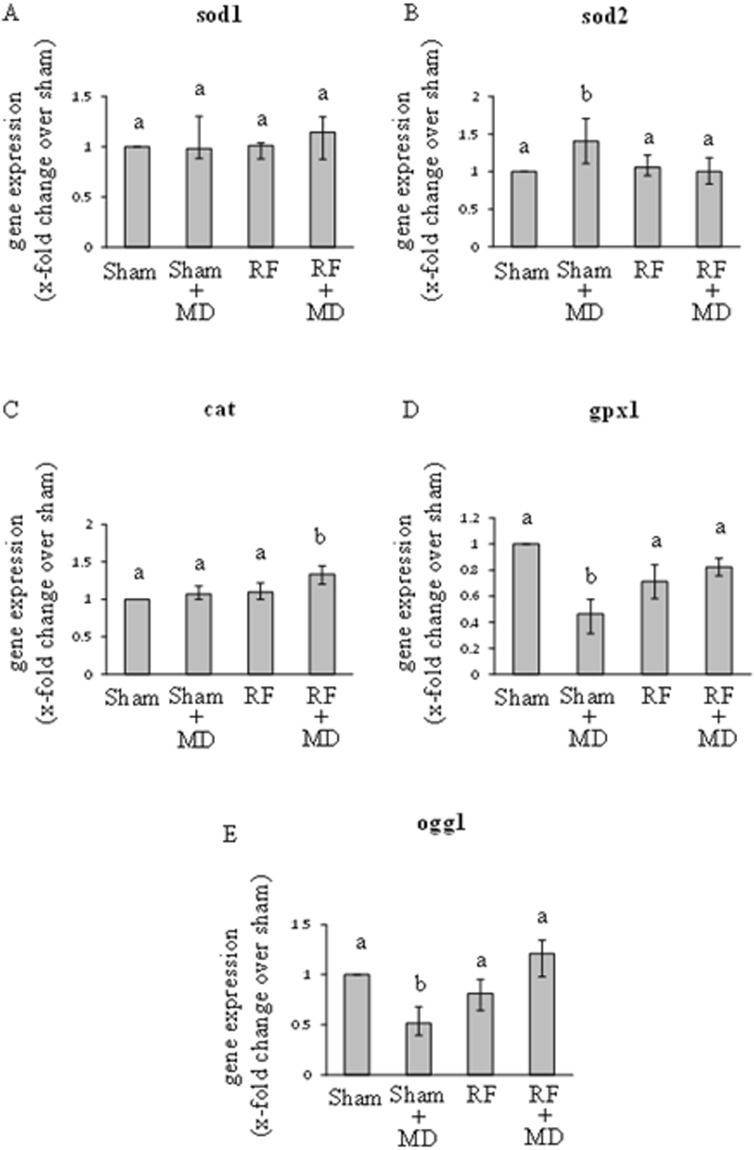
Medians with interquartile ranges of RT-PCR-based gene expression data (2^−ΔΔCt^ method) for antioxidant and DNA repairing enzymes. Sod1 (superoxide dismutase 1, panel A), sod2 (superoxide dismutase 2, panel B), cat (catalase, panel C), gpx1 (glutathione peroxidase 1, panel D), ogg1 (8-oxoguanine DNA glycosylase 1, panel E). Cells sham-exposed for 20 h (Sham); sham-exposed and treated with 10 µM MD for 1 h (Sham + MD); RF-exposed for 20 h at 0.3 W/Kg (RF); RF-exposed for 20 h and treated with 10 µM MD for 1 h (RF + MD). Same letters indicate no statistically significant differences (please, see main text for statistical details).

The results obtained from six experiments are summarized in Fig. 3.

Sod1 transcript level was not affected by either MD or RF. In addition, the rank sum test revealed no difference between Sham + MD and RF + MD conditions.

The rank sum tests found that sod2 transcript level was significantly increased by MD (Sham + MD *vs*. Sham; P < 0.05), whereas sod2 mRNA level was not changed by RF. The analysis revealed also that the pre-exposure of MD-treated cells to RF restored the level of sod2 transcript to that detected in sham condition, as well as in cells exposed to RF alone.

Mann-Whitney U tests found that cat mRNA amount was not affected by either MD or RF. The rank sum test revealed also that the transcript level of catalase was increased only when cells were treated with both RF and MD (P < 0.05 *vs*. Sham + MD).

With regard to glutathione peroxidase 1, the analysis of gene expression showed that the gpx1 transcript was decreased significantly by MD (P < 0.05) but not by RF. Statistics revealed also that RF pre-exposure of MD-treated cells restored the level of gpx1 transcript to that detected in sham-exposed controls.

Similarly, gene expression of ogg1 was decreased significantly by MD (P < 0.05) but not by RF, and RF pre-exposure of MD-treated cells restored the level of ogg1 mRNA to that observed in sham-exposed controls.

## Discussion

The induction of AR as defence mechanism against cytotoxic chemical and physical treatments is a well-documented phenomenon, which has been characterized in different *in vitro* and *in vivo* experimental systems^4,16^.

Over the last years, several studies have been performed by independent research groups describing the ability of non ionizing electromagnetic fields to induce AR *in vitro*, as well as *in vivo*, acting as AD. Most of them refers to RF, but data on extremely low frequencies have also been reported^17–19^. Concerning RF, the phenomenon has been observed both at frequencies and signals in use for telecommunications (900–1950 MHz; continuous wave, GSM, UMTS signals)^1,10,20–23^ and at extremely high frequencies (42.2 GHz)^24^. In all cases, pre-exposure significantly reduced the damage induced by a large variety of chemical and physical agents with different action mechanisms.

The results of our previous studies showed that 20 h pre-exposure of human peripheral blood lymphocytes to RF-EMF, as well as of Chinese hamster lung fibroblasts, significantly reduced the DNA damage induced by subsequent genotoxic treatments with chemical (MMC)^9–12^ and physical (X rays)^13^ agents, as assessed by applying the cytokinesis-block micronucleus assay. The findings reported in the present investigation confirmed these observations in terms of DNA strand breaks in SH-SY5Y, a human neuroblastoma cell line, challenged with MD, a well-known ROS-promoting oxidative stress inducer^15^. In addition, our results suggested the involvement of mechanisms controlling the expression of enzymes responsible for antioxidant defence and DNA-repair. In particular, we revealed that the expression of sod1, the cytoplasmic form of superoxide dismutase, was not affected by either MD or RF treatment. Conversely, the gene expression of SOD2 (alias, Mn-SOD), the mitochondrial form of superoxide dismutase, was changed by treatments. Literature reports that one of the cytoplasmic organelles most involved in the MD cytotoxic action is the mitochondrion^15,25,26^. In particular, our experiments showed that the pro-oxidant action of MD increased the mRNA level of SOD2, which may reflect higher needs to convert O_2_^.−^ to H_2_O_2_ within the mitochondrion^27^. However, we found no significant changes in transcript levels of catalase, the enzyme that scavenges H_2_O_2_^28^. Furthermore, in our experiments MD strongly reduced the gene expression level of GPX1, the GSH-requiring enzyme that reduces organic hydroperoxides and H_2_O_2_^29^. Such a reduction suggests that the MD-related ROS overproduction could be powerful, and that catalase may be involved in ROS detoxification more than glutathione peroxidase, maybe due to its greater Km for H_2_O_2_^30^. In addition, it is known that MD causes a depletion of GSH, which is essential to the catalysis of GPX1^29^, and this may help to explain the reduced transcriptional expression of glutathione peroxidase.

In coherence with its genotoxic activity, our experiments revealed that MD also decreased the transcript level of OGG1, which is an enzyme involved in both nuclear and mitochondrial DNA repair^31^.

Despite the significant changes induced by the pro-oxidant challenge in the transcriptional profile, MD-related DNA damage was not reverted, and this suggests that the response of SH-SY5Y cells to MD treatment was not effective. It should be noted that if not followed by a similar increase in H_2_O_2_-targeting removal, an increase in SOD2-related action leads to an accumulation of hydrogen peroxide, which may diffuse and damage other compartments far from production site^32^. Moreover, in MD-treated cells we observed a significant decrease in ogg1 mRNA, which is likely to be related to a loss of DNA repair capacity, and such a transcriptional pattern is coherent with the increased level of DNA damage following MD treatment.

In our experiments, RF treatment did not elicit any statistically significant effect on SH-SY5Y cells. However, when RF-exposed cells were treated with MD, we observed an adaptive response that reverted the MD effect for many of the endpoints considered. Indeed, RF abolished the MD-related overexpression of Mn-SOD mRNA. Moreover, the co-treatment with RF + MD reverted the effects of MD on both GPX1 and OGG1 transcript levels. The combined treatment also increased the gene expression level of catalase, leading to a possible improvement of the cellular response to oxidative stress and to a reduced genotoxic damage.

It is worth of note that considerations about the efficiency of the antioxidative enzymatic defence should be made taking into account also the ratios of the expression of coupled antioxidant enzymes, rather than the expression of enzymes alone. Indeed, in coupled enzymatic reactions the product of one reaction serves as the substrate for the next reaction. Therefore, the overall transformation of chemicals is ensured by the overall flow through the whole enzymatic pathway. In particular, catalase and glutathione peroxidase ensure the prompt removal of SOD-generated superoxide anions^33^. Therefore, an increase in both GPX/SOD and CAT/SOD ratios is a reliable indicator of the cellular scavenging efficiency against the O_2_^.−^/H_2_O_2_ redox couple^17,34,35^. Our results revealed that MD increased sod2 gene expression, and simultaneously decreased gpx1 transcript level, without affecting significantly the mRNA level of catalase. This clearly indicates that upon treatment with MD, neuroblastoma cells underwent a marked reduction of the both GPX- and CAT-based antioxidative enzymatic efficiency, at least at transcriptional level. In particular, as compared to sham-exposed cells (calibrator = 1.00), Sham + MD cells showed strongly decreased ratios of gpx1/sod1 (0.49), gpx1/sod2 (0.34), and cat/sod2 (0.76), whereas the cat/sod1 ratio resulted only slightly increased (1.09). Our results also indicate that the pre-exposure of neuroblastoma cells to RF strongly limited the MD-dependent decrease in both gpx1/sod1 (0.72) and gpx1/sod2 (0.82) ratios. Moreover, the CAT-based antioxidant efficiency was clearly elevated by RF, as we found that the pre-exposure of neuroblastoma cells to RF completely reverted the MD-induced decrease in cat/sod2 ratio (1.33). Taken together, our transcriptional analyses suggest that RF pre-exposure almost inhibited the dramatic loss of GPX-based antioxidant scavenging efficiency that was induced by MD, and in parallel strongly enhanced the gene expression of CAT-based antioxidant protection. Such results suggest that RF pre-treatment rendered SH-SY5Y cells less susceptible to oxidative stress, likely due to higher ROS scavenging capacities. In future experiments, we will extend our investigations in order to verify whether such regulation of gene expression may be followed by a similar modulation of enzymatic activities.

In addition, RF reverted the MD-induced decline of OGG1 gene expression, and this may imply that RF pre-exposure preserves the cellular capacity to repair oxidatively-damaged DNA. Taken together, our data are in good accord with the absence of any significant induction of genotoxic effect of MD in RF-exposed cells.

The results of the present investigation are in agreement with those reported in the literature by independent researchers, both *in vitro* and *in vivo*.

A decreased number of gamma rays-induced DNA strand breaks and a faster kinetics of DNA repair was reported by Ji *et al*. in mouse bone-marrow stromal cells pre-exposed to 900 MHz at 120 µW/cm^2^ power density^21^. By using the same cell type and the same RF-exposure conditions, the authors also found a significant increase in poly (ADP-ribose) polymerase (PARP) expression and its protein levels when RF-exposed cells were compared to sham-exposed controls^20^.

In a previous study, the same research group also found a reduction of bleomycin (BLM)-induced DNA strand breaks in IRC mice pre-exposed to RF for 4 hours/day for 7 days and then injected with BLM. In addition, they also detected a reduction in BLM-induced malondialdehyde levels and an increment of BLM-induced SOD reduction^23^.

Mortazavi *et al*. pre-exposed Sprague Dawley rats to 915 MHz, GSM, and subsequently to gamma rays. They found that the gamma rays-induced reduction in glutathione reductase and reduced glutathione concentration were restored by RF pre-exposure^22^.

RF-induced AR was also detected at extremely high frequencies. A protection against strand breaks induced by X rays was reported in mouse leukocytes pre-exposed *in vitro* to pulse-modulated 42.2 GHz (1.5 W/kg SAR)^24^.

In conclusion, RF exposure induced an adaptive conditioning of neuro-derived human cells. It reverted the DNA damage induced by MD treatment and abolished the MD-induced impairment in the expression of genes involved in the cellular response to pro-oxidant challenges. Given that in our experiments the RF exposure preceded the incubation with MD, it should be highlighted that the RF-dependent pre-conditioning persisted also when the physical factor was removed, thus indicating that RF-induced changes required the permanent modification of existing factors, or the formation of long-lived biochemical or molecular mediators. Lastly, the experimental evidence of different participation of SOD1 and SOD2 in the RF-evoked cytoprotective effect emphasizes the possibility that mitochondria may be strictly involved in the adaptive response evoked in cells by RF exposure. This could pave the way to future implementation of RF-based treatments to modulate the mitochondrial redox milieu, which is emerging as a crucial determinant of cellular health under both physiological and pathological conditions^36–38^.

## Material and Methods

### Exposure system set up and dosimetry

The exposure of SH-SY5Y cell cultures to 1950 MHz, UMTS electromagnetic field was completed by means of a well-established set-up, specifically designed and realized to gain highly controlled electromagnetic and environmental conditions, as widely reported in our previous papers^10,12,39^.

**Figure 4:**
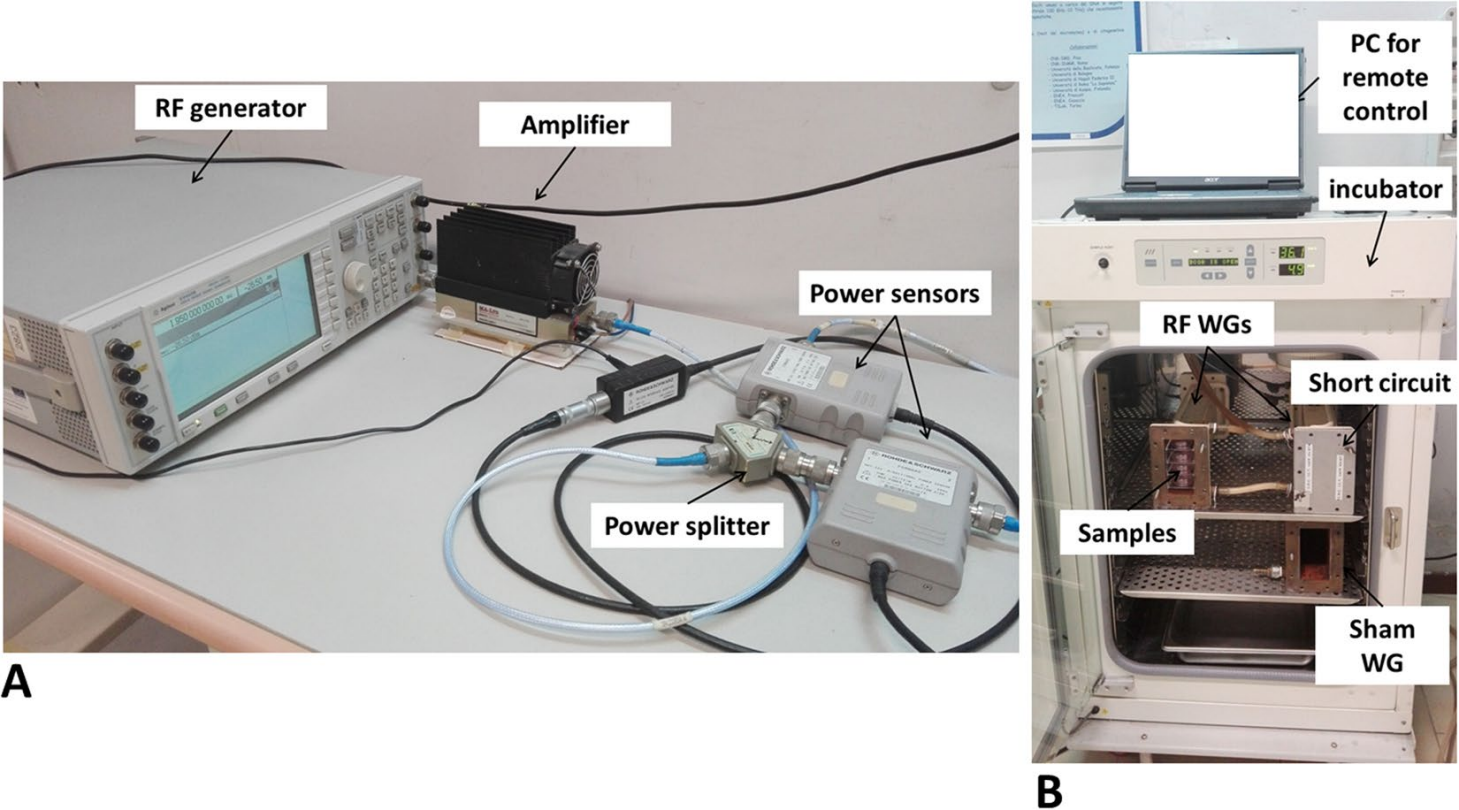
A picture of the experimental set-up used for the exposure of cell cultures to RF. (**A**) Signal generation and conditioning side; (**B**) RF application side (WG stands for waveguide).

A picture of the exposure set-up is shown in Fig. 4. The signal generation and conditioning side is in panel (a), while RF applicators loaded with biological samples are in panel (b). In particular, the 1950 MHz, UMTS signal was provided by a RF generator (E4432B ESG-D, Agilent, Santa Clara, CA), sent to an amplifier (AM38A-0925-40-43; Microwave Amplifier, Bristol, England), and then to a 6 dB power splitter (HP11667A, Hewlett-Packard, Palo Alto, CA). In such a way, two identical signals were obtained and sent, through a couple of bidirectional power sensors (NRT-Z43, Rohde & Schwarz, Munich, Germany), to two identical RF applicators consisting of rectangular, short-circuited waveguides (WG, WR430, 350 mm long, SAIREM, Neyron, France), connected to the feeding side by means of a coaxial-to-waveguide adapter (Maury Microwave R213A2, VSWR: 1.05, Montclair, CA). Both the signal generator and the power sensors were remotely controlled by a PC in a feedback loop, that was employed to continuously monitor the incident and reflected power levels, and adjust them to keep the required SAR constant.

The two waveguides were placed inside a cell culture incubator (to guarantee a 37 °C, 95% air and 5% CO_2_ atmosphere), together with a third, identical one, disconnected from the RF feeding and used for sham-exposures.

Four samples of SH-SY5Y cultures were exposed at the same time in each short-circuited waveguide by means of a four-layer Plexiglas stand. The position of samples inside the waveguides was optimized through numerical dosimetry simulations, to maximize exposure efficiency and electric field uniformity^10,12,13^. In such simulations, the rectangular waveguide (inner transversal dimensions: 109.2 mm × 54.6 mm) was modelled as perfectly conductive with the longitudinal axis and narrow side horizontal, to achieve the condition of an unperturbed (empty waveguide) E vector, parallel to the sample layer. The four samples were modelled as cylinders (diameter 34 mm, thickness 3.3 mm) with electromagnetic properties of cell culture medium (relative permittivity, ε_r_, 75, effective conductivity, σ, 2.2 S/m, and mass density, ρ, 1060 kg/m^3^), placed inside four polystyrene (ε_r_, 2.6), 35 mm Petri dishes and located on a four-layer Plexiglas stand. It was demonstrated that, under the TE10 propagation mode, by placing the sample centres at a distance of 0.56 λ_z_ from the short-circuit, efficiency of the exposure system was approximately 70%, and the degree of non-uniformity of SAR distribution was 0.33 in all samples. Moreover, by exploiting the parabolic profile of the electric field in the waveguide cross-section, the vertical distance between samples was arranged in such a way to simultaneously expose two couples of samples to two different SAR values^40^.

For experiments devoted to evaluate the effect of RF exposure by applying the comet assay, in cell cultures exposed to 0.3 and 1.25 W/kg SAR, the above described set-up was adopted with one waveguide loaded with cell cultures and the other with dummy cultures.

For experiments aimed at measuring gene expression, the above exposure set up was adopted with both waveguides loaded with cell cultures in the distal positions (0.3 W/kg SAR) and dummy cultures in the central positions.

To exclude any RF heating effect during exposures, temperature measurements were carried out at regular 5-s intervals for 20 h (accuracy of ±0.3 °C) in separate experiments, using a fiber-optic thermometer (FisoUMI4, FISO Technologies, Quebec, Canada) with a fiber-optic temperature probe (FISO Technologies, FOT-M/2 m) inserted horizontally into the culture medium. In five independent measurements the temperature never exceeded the instrument sensitivity (±0.3 °C).

### Reagents

Dulbecco’s modified Eagle’s medium (DMEM), foetal bovine serum (FBS), L-glutamine, trypsin EDTA and penicillin/streptomycin were from Biowhittaker (Verviers, Belgium). Triton X-100, N-lauryl sarcosine and menadione were from Sigma (St. Louis, MO). Dimethyl sulfoxide, NaOH and Na_2_EDTA were from Baker (Deventer, The Netherlands). Tris and trypan blue were from BDH (Poole, England); NaCl was from Carlo Erba (Milan, Italy). Normal-melting-point agarose, low-melting-point agarose and ethidium bromide were from Bio-Rad Laboratories (GmbH, Munich, Germany). Ribospin kit and Riboclear plus were purchased by GeneAll Biotechnology CO, Ltd (Seoul, Korea). cDNA was obtained via reverse transcription (by OriGeneTechnologies, Inc. (Rockville, MD, USA). SensiFast SYBR-based kit was from Bioline (London, UK). Custom primers were synthetized by IDT Integrated DNA Technologies, Inc. (Coralville, IA, USA).

### Cell cultures and maintenance

Human SH-SY5Y neuroblastoma cell line was a gift of Prof. P. Abrescia (University of Naples, Italy). Cells were cultured in DMEM, supplemented with 10% heat-inactivated FBS, 2 mM L-glutamine, 100 U/ml penicillin, and 100 mg/ml streptomycin. Cells were hosted in a commercial incubator (model 311, Forma Scientific, Freehold, NJ, USA) at 37 °C in an atmosphere of 95% air and 5% CO_2_. Cells were supplied with fresh culture medium every 48 h and kept exponentially growing by splitting them once a week by tripsinization (five minutes treatment with 200 mg/ml trypsin-EDTA solution).

### Experimental procedures

For consistency and reproducibility, a master bank of cells was established and the same batch of reagents was used. Each experimental run was carried out on cells harvested from the same parent flask, and 48 h before the experiments, 3 ml cultures were set up by seeding 10^5^ cells in 35 mm coded Petri dishes (Corning, catalogue no. 430165, New York, NY), and grown for a total of 72 h.

Three MD concentrations were tested to identify the most suitable to act as CD in the alkaline comet assay. To this aim, MD (1 mg/ml in DMSO, dissolved immediately before treatments), was given 1 h before cell harvest (h 71–72) at 5, 10 and 20 µM final concentration.

To test the responsiveness of SH-SY5Y cells in exhibiting AR in terms of reduction of MD-induced DNA damage, five experiments were carried out using MD for both AD (0.05 µM, added at 48 h after seeding) and CD (10 µM, added at 71 h) treatments. Both AD and CD were left throughout the culture period, i.e. until 72 h of growth.

The capability of RF-EMF to induce AR in terms of primary DNA damage was evaluated in cell cultures pre-exposed for 20 h to 0.3 W/kg and 1.25 W/kg SAR values. Each experimental run included eight conditions: control (incubator), sham-exposure, RF exposure at 0.3 W/kg (AD), RF exposure at 1.25 W/kg (AD), MD 10 µM (CD) given 71 h after seeding, sham-exposure +MD, RF 0.3 W/kg (AD) + MD (CD), and RF 1.25 W/kg (AD) + MD (CD).

In order to investigate gene expression of DNA repairing and antioxidant enzymes, 20 h pre-exposure to RF was carried out at 0.3 W/kg. For each experiment, four conditions were considered: sham-exposure, sham-exposure +MD, RF exposure (AD), and RF (AD) + MD (CD).

**Figure 5:**
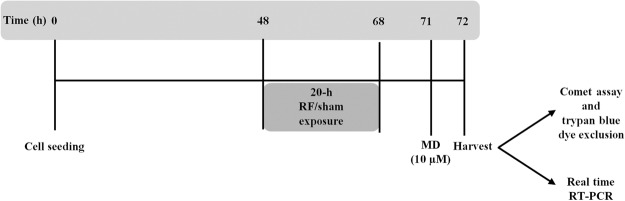
Schematic representation of the experimental procedure adopted to expose human neuroblastoma SH-SY5Y cells to 1950 MHz, UMTS signal. After 48 h of growth, treatment with radiofrequency (RF)/sham was given for 20 hours, alone or in combination with 10 µM menadione (MD) added at 71 h. 72 hours after seeding, cells were harvested and processed to evaluate viability, by trypan blue dye exclusion assay, primary DNA damage, by alkaline comet assay, or gene expression, by real time RT-PCR.

The experimental procedure above described is presented in Fig. 5.

### Trypan blue dye exclusion assay

The method is based on the principle that trypan blue enters only dead cells via damaged cell membrane. Just prior to counting, cell aliquots of 10 µl were mixed with 10 µl of trypan blue staining solution (0.4% w/v). Aliquots of 10 µl of the mixture were loaded on a Luna counting slide and the number of viable cells (unstained) was determined using the Luna II automated cell count (Logos Biosystems, Inc., Anyang-City, South Korea), an image-based cell counting device for cell counting and viability analysis. The device provides the total number of cells per ml and the number of viable and dead cells.

### Alkaline comet assay

The method developed by Singh and co-workers was employed^41^, with further modifications to obtain a consistent DNA migration in the control cells and a subsequent higher sensitivity^42^. After trypsinization, cells were collected and centrifuged for 5 min at 400 g. Cell viability was assessed using the trypan blue exclusion method. Aliquots of about 7 × 10^4^ viable cells were centrifuged for 10 min at 800 g. The supernatant was discarded and cells, suspended in 100 µl low-melting point agarose (0.5% w/v), were sandwiched between a lower layer of 1% normal-melting agarose at 37 °C and an upper layer of low melting point agarose (0.6% w/v) on microscope slides. The slides were then immersed for 60 min in a freshly prepared cold lysing solution made by 2.5 M NaCl, 100 mM Na_2_EDTA, 10 mM Tris, pH 10, 1% N-lauryl sarcosine, with 1% triton X-100 and 10% DMSO at 4 °C added just before use. At the end of lysis treatment, slides were drained and placed in a horizontal gel electrophoresis tank with fresh alkaline electrophoresis buffer (300 mM NaOH, 1 mM Na_2_EDTA, pH 13) and left in the solution for 40 min at 4 °C to allow the equilibration and DNA unwinding to express alkali labile damage. Using the same buffer, electrophoresis was carried out at 4 °C for 40 min at 30 V by using an Amersham Pharmacia Biotech power supply (Uppsala, Sweden) and adjusting the current to 340 mA by modulating the buffer level. Then, slides were rinsed three times with Tris (400 mM, pH 7.5) and left in distilled water for 5 min. Slides were air-dried and stained just before analysis with 12 μg/ml ethidium bromide. For each condition, 2 slides were set up and images of 500 randomly selected nuclei (250 from each slide) were analysed using a computerized image analysis system (Delta Sistemi, Rome, Italy) fitted with a Leica DM BL fluorescence microscope (Leica Microsystems, Mannheim, Germany) at 200X magnification. This system acquires images and evaluates a range of derived parameters. DNA integrity was evaluated taking into account the percentage of migrated DNA in the tail and tail moment^43^. Data were decoded after completion of all microscopic analyses.

### RNA extraction and real time RT-PCR analysis

RNA was extracted by using Ribospin kit and contaminant DNA was degraded by Riboclear plus, following the kit supplier’s recommendations. The total RNA concentration and purity were estimated by measuring UV absorbance at 260/280/320 nm using a Lambda25 spectrophotometer (PerkinElmer Inc., Waltham, MA, USA). The resulting RNA (1 µg) was used to obtain cDNA via reverse transcription. The cDNA was used for the SYBR-based PCR step, diluting the sample 1:100 for both cat and sod1, 1:10 for both sod2 and gpx1, and 1:50 for ogg1, by using an Applied Biosystems 7300 thermalcycler (ThermoFisher Scientific, Inc., Rockford, IL, USA). Custom primers were synthetized by IDT Integrated DNA Technologies, Inc. (Coralville, IA, USA). The list of primers used, along with the corresponding references, was reported in Table 1. Amplification steps were set as follows: 95 °C for 5 sec, 60 °C for 30 sec (40 cycles), after an initial denaturation at 95 °C for 2 min, as recommended by supplier. The formation of unspecific amplicons was verified by performing final melting curve analyses for each primer pair used (95 °C for 15 sec, 60 °C for 1 min, 95 °C for 15 sec, and 60 °C for 15 sec).The ΔΔCt method was used for the determination of relative expression, using β-actin and sham sample as reference mRNA and calibrator sample, respectively^44^. All samples were processed by analysing results from six independent experiments.

**Table 1:** Primer pairs used for real time RT-PCR-based amplifications and the corresponding references.

Transcript	Fw 5′ → 3′	Rv 5′ → 3′	References
β-actin	ATTGCCGACAGGATGCAGA	AGTACTTGCGCTCAGGAGGA	^42^
cat	AGGGGCCTTTGGCTACTTTG	ACCCGATTCTCCAGCAACAG	^43^
sod1	AGGCATGTTGGAGACTTGGG	CCACAAGCCAAACGACTTCC	*
sod2	AGGCTCAGGTTGGGGTTGGCT	GCGTGCTCCCACACATCAATCCC	^44^
gpx1	GGACTACACCCAGATGAACG	TCTCTTCGTTCTTGGCGTTC	^45^
ogg1	GCATCGTACTCTAGCCTCCA	GCTCTTGTCTCCTCGGTACA	^46^

*Primer pair (Fw, forward; Rv, reverse) obtained by using Prime Blast, NM_000454.4.

### Statistical analysis

Data were expressed as medians with interquartile ranges, and statistical analysis of data was performed by StatSoft, Inc. (2011). STATISTICA (data analysis software system), version 10. www.statsoft.com. Non-parametric analyses were performed by applying Mann-Whitney U tests. P lower than 0.05 was considered as statistically significant.

## Data Availability

All data generated or analysed during this study are available from the corresponding author on reasonable request.
